# Evaluating the Role of *BglI* rs739837 and *TaqI* rs731236 Polymorphisms in Vitamin D Receptor with SARS-CoV-2 Variants Mortality Rate

**DOI:** 10.3390/genes13122346

**Published:** 2022-12-12

**Authors:** Wisam Hasan Madhloom Albu-Mohammed, Enayat Anvari, Abolfazl Fateh

**Affiliations:** 1 Department of Biology, Science and Research Branch, Islamic Azad University, Tehran 1477893855, Iran; 2 Department of Physiology, School of Medicine, Ilam University of Medical Science, Ilam 693917134, Iran; 3 Department of Mycobacteriology and Pulmonary Research, Pasteur Institute of Iran, Tehran 1316943551, Iran; 4 Microbiology Research Center (MRC), Pasteur Institute of Iran, Tehran 1316943551, Iran

**Keywords:** Vitamin D receptor, coronavirus disease 2019, severe acute respiratory syndrome coronavirus 2 variants

## Abstract

A lack of vitamin D is a potential risk factor for coronavirus disease (COVID-19). Variants in the Vitamin D Receptor (*VDR*) gene, such as *Bgl*I rs739837 and *TaqI* rs731236, are associated with various viral infection progressions. This study aimed to evaluate the relationship between the *BglI* rs739837 and *TaqI* rs731236 polymorphisms and the mortality rate of COVID-19 based on severe acute respiratory syndrome coronavirus 2 (SARS-CoV-2) variants. The genotyping of *BglI* rs739837 and *TaqI* rs731236 genotypes was analyzed using the polymerase chain reaction–restriction fragment length polymorphism in 1734 improved and 1450 deceased patients positive for SARS-CoV-2. In this study, the rate of COVID-19 mortality was correlated with *TaqI* rs731236 TC and CC in the α variant and with *TaqI* rs731236 CC in the Delta variant, whereas no relationship was found in the Omicron BA.5 variant. In addition, the rate of COVID-19 mortality was associated with *BglI* rs739837 GT and TT in the Omicron BA.5 variant, while there was no association between *BglI* rs739837 and COVID-19 mortality in the α and Delta variants. The TG haplotype was more common in all SARS-CoV-2 variants, while the CT haplotype was associated with COVID-19 mortality in the Delta and Omicron BA.5 variants. In conclusion, this study indicated that the impacts of *BglI* rs739837 and *TaqI* rs731236 polymorphisms were related to SARS-CoV-2 variants. However, further research is still needed to approve our findings.

## 1. Introduction

The coronavirus disease (COVID-19) pandemic has reconcentrated attention on strategies to hamper acute respiratory infections. The mainstay of disease control is vaccination against severe acute respiratory syndrome coronavirus 2 (SARS-CoV-2), although this approach is not always practical worldwide due to issues including cost, availability, vaccine reluctance, vaccine failure, and vaccine escape. Complementary, low-cost methods are required to boost immunity to SARS-CoV-2 and other viruses that cause acute respiratory tract illnesses [1,2,3].

The World Health Organization (WHO) identified the SARS-CoV-2 variants α, β, γ, Delta, and Omicron as the sources of concern during the pandemic. However, the α and Delta variants are more contagious and cause more deaths than the others [4]. It is currently unclear how human genomic variations and the severity of COVID-19 interact. Early research suggested a link between COVID-19 and a deficiency of 25-hydroxyvitamin D [5].

Long recognized as supporting innate immune responses to respiratory bacteria and viruses, the metabolites of 25-hydroxyvitamin D control immunopathological inflammation [6,7]. Cathelicidin LL-37 and human β defensin 2 are 25-hydroxyvitamin D–inducible antimicrobial peptides that bind to the SARS-CoV-2 spike protein and prevent viral binding to the cellular receptor angiotensin converting enzyme 2 (ACE2) [8]. Several bodies of research examining possible links between higher 25-hydroxyvitamin D status or vitamin D supplement use and decreased risk of SARS-CoV-2 infection have produced conflicting findings, with some indicating protective relationships and others indicating null or adverse associations [9,10,11,12,13]. Generally, meta-analyses that include these and other observational research indicate protective relationships [14,15].

Vitamin D’s biological effects are mediated via its nuclear receptor, which serves as a ligand-activated transcription factor [16]. Even though the Vitamin D receptor (VDR) is abundantly expressed in lung tissue, the possible significance of vitamin D–VDR signaling in pulmonary immunopathology is unknown [17]. The highly polymorphic *VDR* gene encodes VDR. The *VDR* gene single nucleotide polymorphisms (SNPs) that are most frequently described include *Apa*I (rs7975232; intron 8; C > A), *Bsm*I (rs1544410; intron 8; G > A), *Fok*I (rs2228570; exon 2; C > T), *TaqI* (rs731236; exon 9; A > G), *EcoRV* or A-1012G/GATA (rs4516035; promoter; T > C), and CDX2 (rs11568820; promoter; G > A), which were correlated with vitamin D secretion. Previous research discovered that these SNPs are linked to various inflammatory illnesses, including community-acquired pneumonia [18], dengue [19], and COVID-19 [20].

The VDR gene’s 3′-untranslated region (UTR) contains the *Bgl*I (rs739837; 3′UTR region; C > T) polymorphism, which may impact the posttranscriptional regulation of the *VDR* gene via interacting with microRNA (miRNA). MicroRNAs play a crucial role in the control of gene expression; hence, SNPs in the seed sites of miRNA targets can generate or eliminate miRNA-binding sites, thereby influencing phenotypes and disease susceptibility [21,22]. *Taq1* rs731236 is located in the exon 9 of the VDR gene and produces a synonym change of the coding sequence; therefore, it does not generate an amino acid change of the encoded protein, but it might affect the stability of the mRNA [23]. Data on the association of VDR polymorphism (*Taq1* rs731236) with tubercular meningitis and pulmonary tuberculosis revealed that this polymorphism was associated with these diseases. Associations between the *Taq1* rs731236 and the development of diseases such as chronic hepatitis B virus infection, liver disease progression, and acute lower respiratory tract infection (ALRI) have also been reported [24,25,26].

The current study investigated the relationship between the *BglI* rs739837 and *TaqI* rs731236 polymorphisms and the COVID-19 mortality rate based on SARS-CoV-2 variants.

## 2. Materials and Methods

### 2.1. Sample Collection

From November 2020 to February 2022, patients referred to the Ilam University of Medical Sciences were studied retrospectively at three peaks of COVID-19 infection (α, Delta, and Omicron BA.5).

Pharyngeal swab samples were analyzed using real-time reverse transcription polymerase chain reaction (rtReal time–PCR) to identify the SARS-CoV-2 genome. The identical parameters for sample time, the RNA extraction kit, and the real-time PCR kit were used for all rtReal time–PCR experiments conducted by qualified individuals.

According to the following inclusion criteria, only 3184 out of 14,117 patients were considered eligible to participate in the study: (1) giving consent before participating in the study, (2) sharing the same ethnic background and Iranian nationality, (3) having positive rtReal time–PCR test results and being chosen from only one hospital, and (4) not having any underlying comorbidities such as diabetes, liver disease, obesity, cystic fibrosis, chronic obstructive pulmonary disease, kidney disease, cancer, heart disease, pregnancy, or human immunodeficiency virus (HIV).

The study patients were divided into two groups. Group 1 contained patients who had improved with a wide range of symptoms (e.g., loss of taste and smell, sore throat, fever, cough, malaise, nausea, headache, vomiting, diarrhea, and muscle pain). Some had a lower respiratory illness during clinical evaluation or imaging, with oxygen saturation (SpO2) below 94% on room air at sea level. Most patients with minor illnesses were treated in an outpatient setting or at home via telemedicine or telephone visits.

Group 2 contained deceased patients with the following characteristics: patients with COVID-19 with SpO_2_ of 94% on room air at sea level, PaO_2_/FiO_2_ of 300 mm Hg, a respiratory rate >30 breaths/min, or lung infiltrates >50%, multiple organ dysfunction, and septic shock, all of whom were deemed to have severe disease.

All of the clinical parameters of the patients were analyzed as soon as they were admitted to the hospital. They included cholesterol, triglyceride (TG), high-density lipoprotein (HDL), low-density lipoprotein (LDL), white blood cells (WBC), platelets, erythrocyte sedimentation rate (ESR), C-reactive protein (CRP), uric acid, creatinine, fasting blood glucose (FBS), real-time PCR cycle threshold (Ct) values, 25-hydroxyvitamin D, aspartate aminotransferase (AST), alkaline phosphatase (ALP), and alanine aminotransferase (ALT).

### 2.2. BglI rs739837 and TaqI rs731236 Genotyping

The High-pure PCR Template Preparation Kit (Roche Diagnostics Deutschland GmbH, Mannheim, Germany) isolated genomic DNA from the blood samples, according to the manufacturer’s instructions. The purity and quality of DNA were evaluated using NanoDrop spectrophotometers (Thermo Scientific, USA) and gel electrophoresis, respectively.

The polymerase chain reaction–restriction fragment length polymorphism (PCR-RFLP) assay was used for *BglI* rs739837 and *TaqI* rs731236 genotyping. The forward and reverse primers for *BglI* rs739837 were 5’-CACCCAGCCCATTCTCTCTC-3’ and 5’-GCAGGTGTCTCTGTCCCTGA-3’, respectively. The forward and reverse primers for *TaqI* rs731236 were 5’-CCCATGAAGCTTAGGAGGAA-3’ and 5’-TCATCTTGGCATAGAGCAGGT-3’, respectively. The PCR product sizes were 248 bp and 699 bp, respectively. For both SNPs, the PCR test was performed by initial denaturation at 94 °C for 5 min, followed by 35 cycles of 94 °C for 30 s, 60 °C for 30 s, 72 °C for 45 s, and final extension at 72 °C for 10 min.

The PCR products were digested with *BglI* and *TaqI* restriction enzymes (Fermentas, Vilnius, Lithuania) for *BglI* rs739837 and *TaqI* rs731236 at 37 °C for 16 h, respectively. After incubation, the RFLP product was visualized by electrophoresis on 2.5% agarose gel. The product sizes were 178 bp and 70 bp for the CC genotype and 248 bp for the TT genotype in *BglI* rs739837, and 699 bp for the TT genotype and 604 bp and 95 bp for the CC genotype in *TaqI* rs731236 [27]. In order to confirm the results of PCR–RFLP, at least 10% of samples were randomly genotyped using the Sanger sequencing method on an ABI 3500 DX Genetic Analyzer (ABI, Thermo Fisher Scientific, Waltham, MA, USA). MEGA Version 11.0 was used to assess the results (https://www.megasoftware.net/ accessed on 23 April 2021).

### 2.3. Statistical Analyses

Using SPSS version 22.0 (SPSS, Inc, Chicago, IL, USA), the categorical data were summarized as numbers (%) and the continuous data as the mean ± standard deviation (SD). The Shapiro–Wilk test was conducted to determine whether numerical variables follow a normal distribution. The chi-square and Mann–Whitney U tests were used to compare qualitative and quantitative data between paired groups of COVID-19 and SARS-CoV-2 variants and the mortality rate between improved and deceased groups with *BglI* rs739837 and *TaqI* rs731236 genotypes and clinical parameters. The independent determinants of COVID-19 mortality and likelihood were found using multivariate models based on logistic regression. Ninety-five percent confidence intervals (CIs) and odds ratios (ORs) were also computed. A *p*-value less than 0.05 was considered statistically significant, and all tests were conducted with two-tailed distributions.

The Hardy–Weinberg equilibrium (HWE), linkage disequilibrium, four inheritance models (dominant, codominant, overdominant, and recessive), and the minor allele frequency (MAF) of the selected variant were calculated using the online SNPStats program. The Akaike Information Criterion (AIC) and the Bayesian Information Criterion (BIC) were used to identify the best model (http://bioinfo.iconcologia.net/SNPStats accessed on 23 May 2006).

## 3. Results

### 3.1. Characteristics of Patients

The characteristics of the COVID-19 study participants are listed in Table 1. In this study, 3184 patients participated, including 1022 α, 1026 Delta, and 1132 Omicron BA.5 variants. The mean age of patients infected with the α, Delta, and Omicron BA.5 variants was 53.0 ± 12.7, 58.0 ± 11.8, and 53.7 ± 12.9, respectively. There were 479 (46.9%) males and 543 (53.1%) females among patients with the α variant and 546 (53.2%) males and 480 (46.8%) females among those with the Delta variant. For the Omicron BA.5 variant, the frequencies of males and females were 546 (53.2%) and 480 (46.8%), respectively. The rate of 25-hydroxyvitamin D was lower in patients with the Delta variant (21.8 ± 10.3) than in those with α (24.2 ± 12.8) and Omicron BA.5 (33.0 ± 13.4) (*p* = 0.029). The mean qPCR Ct values of α, Delta, and Omicron BA.5 were 20.1 ± 6.4, 17.4 ± 6.1, and 21.9 ± 6.0, respectively (*p* < 0.001).

### 3.2. Correlation between TaqI rs731236 and BglI rs739837 Polymorphisms and COVID-19 Mortality Adjusted by SARS-CoV-2 Variants

Patients with the *TaqI* rs731236 CC genotype had a significantly higher COVID-19 mortality rate than those with other genotypes. In addition, COVID-19–recovering patients have the *TaqI* rs731236 TT genotype. In *BglI* rs739837 polymorphism, patients carrying the TT genotype had a higher COVID-19 mortality rate.

The inheritance model analysis results of *TaqI* rs731236 and *BglI* rs739837 polymorphisms among the patients are shown in Table 2. The codominant and overdominant inheritance models with the lowest AIC and BIC values were the best-fitting models for *TaqI* rs731236 and *BglI* rs739837 by comparing the deceased and improved patients, respectively. The *TaqI* rs731236 CC genotype was associated with an increased risk of COVID-19 mortality (*p <* 0.0001, OR 2.01, 95% CI 1.59–2.53), and the *BglI* rs739837 GT genotype was related to a high risk of COVID-19 mortality (*p <* 0.0001, OR 0.52, 95% CI 0.44–0.62).

The *TaqI* rs731236 and *BglI* rs739837 polymorphisms were incompatible with HWE in both improved and deceased patients (*p* < 0.001). It is critical to highlight that HWE may not have been met in the case sample, implying that the SNP is linked to the disease.

### 3.3. TaqI rs731236 and BglI rs739837 Polymorphism Distributions among SARS-CoV-2 Variants

There was a strong link between SARS-CoV-2 variants and the death rate. The high and low mortality rate was shown in the Delta and Omicron BA.5, respectively (*p* < 0.001). In all three variants of SARS-CoV-2, the homozygous TT of *TaqI* rs731236 in individuals was significantly higher than in those with CC or CT genotype (Table 1). After adjusting for SARS-CoV-2 variants and *TaqI* rs731236 genotypes, the rate of COVID-19 mortality was associated with *TaqI* rs731236 TC and CC in the α variant and with *TaqI* rs731236 CC in the Delta variant, whereas no relationship was found in the Omicron BA.5 variant (Table 3).

In all three variants of SARS-CoV-2, the homozygous GG of *BglI* rs739837 in individuals was significantly higher than in those with GT or TT genotype (Table 1). After adjusting for SARS-CoV-2 variants and *BglI* rs739837 genotypes, the rate of COVID-19 mortality was associated with *BglI* rs739837 GT and TT in the Omicron BA.5 variant. There was no association between *BglI* rs739837 and COVID-19 mortality in α and Delta variants (Table 3).

We found that *TaqI* rs731236 and *BglI* rs739837 polymorphisms were in a strong direct linkage disequilibrium (*p* < 0.001), suggesting that risk alleles of these markers are preferably inherited jointly. Based on the results, the TG haplotype was more common in all SARS-CoV-2 variants. The CT haplotype was associated with COVID-19 mortality in the Delta and Omicron BA.5 variants (Table 4).

### 3.4. Factors Related to COVID-19 Mortality

A multivariate logistic regression model investigated the relationship between risk factors and COVID-19 mortality. The COVID-19 mortality rate was correlated with mean age, ALT, HDL, LDL, FBS, uric acid, creatinine, ESR, 25-hydroxyvitamin D, real-time PCR Ct values, SARS-CoV-2 variants, *TaqI* rs731236 CC, and *BglI* rs739837 TT (Table 5).

## 4. Discussion

The current investigation assessed the potential link between COVID-19 susceptibility and *TaqI* rs731236 and *BglI* rs739837 polymorphisms. To the best of our knowledge, this study is the first to show a substantial correlation between COVID-19 mortality in Iran and the *TaqI* rs731236 and *BglI* rs739837 polymorphisms according to SARS-CoV-2 variants.

According to our results, the *TaqI* rs731236 C variant was linked to a higher risk of COVID-19–related death. The MAF (C-allele) for *TaqI* rs731236 in our study was 0.29. This amount was found in South Asian (0.333), East Asian (0.048), Asian (0.050), other Asian (0.056), African (0.285), European (0.395), Latin American (0.239), and African American (0.284) cases, as reported in the NCBI dbSNP database (https://www.ncbi.nlm.nih.gov/snp/TaqI rs731236 accessed on 23 May 2006). The frequency of the *TaqI* rs731236 C-allele was slightly higher in deceased cases (0.34) than improved ones (0.26).

In this study, the MAF (T-allele) for *BglI* rs739837 was 0.24. This amount was reported in South Asian (0.240), East Asian (0.080), Asian (0.070), African (0.516), European (0.486), Latin American (0.201), and African American (0.515) cases, based on the NCBI dbSNP database (https://www.ncbi.nlm.nih.gov/snp/rs739837 accessed on 23 May 2006). The frequency of the *BglI* rs739837 T-allele was slightly higher in deceased cases (0.26) than improved ones (0.22).

Many studies have already assessed the relationship between polymorphisms of the *VDR* gene and susceptibility to COVID-19, and some are in progress [28,29,30]. Earlier studies have shown that active vitamin D has modulatory effects on T cells, including reducing T-cell proliferation, switching responses from T-helper (Th1) to Th2 development, reducing Th17 cell growth, enhancing regulatory cell activity, and reducing inflammatory cytokine production by T-monocytes [23]. All vitamin D regulation functions are dependent on VDR and transporter in an indirect manner [31]. Variations in the SNPs of *VDR* gene could potentially be useful indicators for determining susceptibility to various diseases [32].

In this study, *TaqI* rs731236 CC genotype had a significantly higher COVID-19 mortality rate than did other genotypes. In addition, COVID-19–recovered patients had the *TaqI* rs731236 TT genotype. Our results corroborate with a study that looked at COVID-19 patients in the Cuban population and found evidence of a relationship between SARS-CoV-2 infection and the *TaqI* rs731236 polymorphism in the *VDR* gene [33]. Apaydin et al. indicated that most participants who were admitted to the intensive care unit (ICU) had the TT genotype for *TaqI* rs731236 polymorphism, whereas the TC genotype was more common in the non-ICU–admitted groups [20]. In addition, two studies in Iran did not report any relationship between this polymorphism and the COVID-19 mortality rate [27,34].

The studied patients carrying a TT genotype on the *BglI* rs739837 polymorphism in our study had a higher COVID-19 mortality rate. Abdollahzadeh et al. reported that allelic and genotypic frequencies associated with the *BglI* rs739837 variations did not significantly correlate with clinical symptoms or the severity of COVID-19 [27]. One of the reasons for the difference between our study and others seems to be the sample size. In our study, a much larger number of samples were examined than in these two reports.

*BglI* rs739837, regarded as a silent SNP, is situated in intron 8 at the 3′ end of the VDR gene. These polymorphisms do not alter the encoding protein’s amino acid sequence, but they may influence gene expression by controlling mRNA stability or linkage disequilibrium with other SNPs that influence disease risk [35]. The interaction between vitamin D and VDR may be disrupted by several *VDR* polymorphisms, such as *TaqI* rs731236. As a result, the activity of vitamin D–related signaling pathways may be reduced, leading to an unregulated release of pro-inflammatory cytokines. Numerous studies have shown a direct link between *VDR* polymorphism and increased pro-inflammatory cytokine levels, which contribute to the severity of the disease [36].

We evaluated the putative effects of *TaqI* rs731236 and *BglI* rs739837 polymorphisms on different variants of SARS-CoV-2 infection and the mortality rate. The rate of COVID-19 mortality was correlated with *TaqI* rs731236 TC and CC in the α variant and with *TaqI* rs731236 CC in the Delta variant, whereas no relationship was found in the Omicron BA.5 variant. In addition, the rate of COVID-19 mortality was associated with *BglI* rs739837 GT and TT in the Omicron BA.5 variant, while there was no association between *TaqI* rs731236 and COVID-19 mortality in the α and Delta variants. Our results showed that the TG haplotype was more common in all SARS-CoV-2 variants. The CT haplotype was associated with COVID-19 mortality in the Delta and Omicron BA.5 variants. We assumed that these two SNPs probably behave differently in various SARS-CoV-2 variants. Nevertheless, what mechanism causes this difference is unknown. The mutations in several genes, such as the spike gene, could produce new variants with increased viral infectivity, immune escape potential, and replication [37,38]. A lack of vitamin D can result in the respiratory syndrome by over-activating the pulmonary renin–angiotensin system (RAS). It is now thought that the morbidity and mortality of COVID-19 are directly correlated with the dysregulated RAS pathway [39].

Indeed, it has been determined that the functional host receptor for SARS-CoV-2 variants’ entrance into the alveolar cells is the ACE2 receptor, a component of the RAS pathway [8]. In a rat model of acute lung injury caused by lipopolysaccharides, vitamin D injection elevated the level of mRNA expression of both *VDR* and *ACE2*, suggesting that enhanced expression of *ACE2* and *VDR* played a role in vitamin D protection against acute lung injury [40].

The mortality rate in our study was shown in the Delta variant with lower 25-hydroxyvitamin D compared to other variants, which was statistically significant. In contrast to our study, according to Apaydin et al., there was no connection between serum 25 (OH) D levels and the severity or mortality of COVID-19. However, *VDR* gene polymorphisms were discovered to be independently associated with the severity of COVID-19 and patient survival [20].

This is one of the few studies to evaluate the relationship between SARS-CoV-2 variants and *VDR* gene polymorphisms. Prior studies focused mainly on the correlation between vitamin D levels and COVID-19 illness. Nonetheless, this study did not investigate the association with other important *VDR* gene polymorphisms. In addition, the relationship with these polymorphisms should be investigated in other ethnicities in Iran.

The study limitations included the following: (1) there was a lack of access to healthy people who did not have a history of COVID-19 infection, (2) the results of this study do not cover the entire population of Iran, so other studies should be conducted with different Iranian ethnicities, (3) an accurate designation of viral RNA copy number using qPCR standard curve is needed to accurately evaluate viral load, while we used Ct values, and (4) we only investigated the effects of *TaqI* rs731236 and *BglI* rs739837 polymorphisms in COVID-19 patients infected by three SARS-CoV-2 variants, and this relationship should be investigated in other variants.

In conclusion, our findings revealed significant variations in the genotype distribution of VDR polymorphisms between various SARS-CoV-2 variants. The results also suggested that patients with *TaqI* rs731236 and *BglI* rs739837 polymorphisms might be more likely to contract SARS-CoV-2 variants and provided evidence supporting the recommendation of vitamin D supplements for people with vitamin D deficiency or insufficiency during or after the COVID-19 pandemic.

## Figures and Tables

**Table 1:** Comparison of laboratory parameters between SARS-CoV-2 variants.

	SARS-CoV-2 Variants			
Variables	α (n = 1022)	Delta (n = 1026)	Omicron BA.5 (n = 1136)	*p*-Value
Deceased/Improved patients	479/543 (46.9/53.1%)	674/352 (65.7/34.3%)	279/839 (24.6/75.4%)	<0.001 *
Mean age ± SD	53.0 ± 12.7	58.0 ± 11.8	53.7 ± 12.9	0.128
Gender (male/female)	525/479 (51.4/48.6%)	546/480 (53.2/46.8%)	598/538 (52.6/47.4%)	0.692
ALT, IU/L (mean ± SD) (Reference range: 5–40)	38.5 ± 24.8	40.8 ± 24.7	35.8 ± 24.2	0.001
AST, IU/L (mean ± SD) (Reference range: 5–40)	34.9 ± 15.5	34.5 ± 14.0	31.9 ± 14.4	<0.001 *
ALP, IU/L (mean ± SD) (Reference range: up to 306)	190.2 ± 84.7	188.6 ± 74.0	177.2 ± 83.5	<0.001 *
Cholesterol, mg/dL (mean ± SD) (Reference range: 50–200)	116.1 ± 34.1	120.5 ± 40.5	123.1 ± 39.4	<0.001 *
TG, mg/dL (mean ± SD) (Reference range: 60–165)	124.1 ± 54.9	121.6 ± 48.8	126.9 ± 55.9	0.245
LDL, mg/dL (mean ± SD) (Reference range: up to 150)	82.8 ± 45.1	85.3 ± 45.3	104.7 ± 48.3	<0.001 *
HDL, mg/dL (mean ± SD) (Reference range: >40)	32.5 ± 11.3	32.1 ± 11.5	33.6 ± 11.7	0.039 *
WBC, 10^9^/L (mean ± SD) (Reference range: 4000–10000)	7627.3 ± 2843.2	7599.2 ± 2715.7	7704.9 ± 2807.7	0.297
CRP, mg/L (mean ± SD) (Reference range: <10 mg/L Negative)	61.6 ± 21.5	63.9 ± 22.0	60.2 ± 21.7	0.122
ESR, mm/1st h (mean ± SD) (Reference range: 0–15)	50.1 ± 16.0	52.3 ± 16.0	49.1 ± 16.1	0.025
FBS, mg/dL (mean ± SD) (Reference range: 70–100)	107.1 ± 41.6	109.8 ± 43.2	106.5 ± 40.7	0.716
Platelets × 1000/cumm (mean ± SD) (Reference range: 140,000–400,000)	184 ± 71	185 ± 74	184 ± 69	0.994
Uric acid, mg/dL (mean ± SD) (Reference range: 3.6–6.8)	4.8 ± 1.8	4.4 ± 1.7	5.2 ± 1.8	<0.001 *
Creatinine, mg/dL (mean ± SD) (Reference range: 0.6–1.4)	0.9 ± 0.3	1.0 ± 0.3	0.8 ± 0.3	<0.001 *
qPCR Ct value	20.1 ± 6.4	17.4 ± 6.1	21.9 ± 6.0	<0.001 *
25-hydroxyvitamin D, ng/mL (mean ± SD) (Sufficiency: 21–150)	24.2 ± 12.8	21.8 ± 10.3	33.0 ± 13.4	0.029 *
*TaqI* rs731236				<0.001 *
TT	560 (54.8%)	542 (52.8%)	616 (54.2%)	
TC	313 (30.5%)	329 (32.1%)	415 (36.5%)	
CC	149 (14.7%)	155 (15.1%)	105 (9.3%)	
*BglI* rs739837				<0.001 *
GG	632 (61.8%)	533 (51.9%)	769 (67.7%)	
GT	304 (29.8%)	441 (43.0%)	218 (19.2%)	
TT	86 (8.4%)	52 (5.1%)	149 (13.1%)	

ALT, alanine aminotransferase; AST, aspartate aminotransferase; ALP, alkaline phosphatase; TG, triglyceride; LDL, low-density lipoprotein; HDL, high-density lipoprotein; WBC, white blood cells; CRP, C-reactive protein; ESR, erythrocyte sedimentation rate; FBS, fasting blood glucose; SD, standard deviation; SARS-CoV-2, severe acute respiratory syndrome coronavirus 2. * Statistically significant (<0.05).

**Table 2:** *TaqI* rs731236 and *BglI* rs739837 polymorphisms association with COVID-19 mortality adjusted by SARS-CoV-2 variants.

*TaqI* rs731236		Groups					
Model	Genotype	Improved Patients	Deceased Patients	OR (95% CI)	*p*-Value	AIC	BIC
Allele	T	2578 (74.0%)	1915 (66.0%)	-	-	-	-
	C	890 (26.0%)	985 (34.0%)	-	-	-	-
Codominant	T/T	1007 (58.1%)	711 (49.0%)	1.00	<0.0001 *	4009.3	4039.7
	T/C	564 (32.5%)	493 (34.0%)	1.32 (1.12–1.55)			
	C/C	163 (9.4%)	246 (17.0%)	2.01 (1.59–2.53)			
Dominant	T/T	1007 (58.1%)	711 (49.0%)	1.00	<0.0001 *	4018.9	4043.1
	T/C-C/C	727 (41.9%)	739 (51.0%)	1.48 (1.28–1.72)			
Recessive	T/T-T/C	1571 (90.6%)	1204 (83.0%)	1.00	<0.0001 *	4018.1	4042.4
	C/C	163 (9.4%)	246 (17.0%)	1.81 (1.45–2.26)			
Overdominant	T/T-C/C	1170 (67.5%)	957 (66.0%)	1.00	0.082	4042.7	4067.0
	T/C	564 (32.5%)	493 (34.0%)	1.15 (0.98–1.35)			
Minor allele frequency (C)		0.29	0.34	-	-	-	-
***BglI* rs739837**							
Allele	G	2576 (74.0%)	2255 (78.0%)	-	-	-	-
	T	892 (26.0%)	645 (22.0%)	-	-	-	-
Codominant	G/G	1006 (58.0%)	928 (64.0%)	1.00	<0.0001 *	3990.1	4020.4
	G/T	564 (32.5%)	399 (27.5%)	1.52 (0.44–0.62)			
	T/T	164 (9.5%)	123 (8.5%)	0.98 (0.75–1.28)			
Dominant	G/G	1006 (58.0%)	928 (64.0%)	1.00	<0.0001 *	4006.1	4030.3
	G/T-T/T	728 (42.0%)	522 (36.0%)	0.61 (0.52–0.71)			
Recessive	G/G-G/T	1570 (90.5%)	1327 (91.5%)	1.00	0.021 *	4044.1	4068.4
	T/T	164 (9.5%)	123 (8.5%)	1.19 (0.91–1.54)			
Overdominant	G/G-T/T	1170 (67.5%)	1051 (72.5%)	1.00	<0.0001 *	3988.1	4012.4
	G/T	564 (32.5%)	399 (27.5%)	0.52 (0.44–0.62)			
Minor allele frequency (T)		0.22	0.26	-	-	-	-

COVID-19, coronavirus disease; OR, odds ratios; CI, confidence intervals; AIC, Akaike information criterion; BIC, Bayesian information criterion. * Statistically significant (<0.05).

**Table 3:** Association between *TaqI* rs731236 and *BglI* rs739837 genotypes and mortality in COVID-19 patients stratified by SARS-CoV-2 variants.

**Variants**	**rs731236 Genotypes**	**Improved Patients**	**Deceased Patients**	**OR (95% CI)**
α	T/T	394	166	1.00
	T/C	112	201	4.26 (3.18–5.71)
	C/C	37	112	7.18 (4.75–10.86)
Delta	T/T	101	158	1.00
	T/C	14	98	1.27 (0.95–1.70)
	C/C	115	396	1.62 (1.09–2.41)
Omicron BA.5	T/T	697	7	1.00
	T/C	3	55	-
	C/C	2	22	-
**Variants**	**rs739837 Genotypes**	**Improved patients**	**Deceased patients**	**OR (95% CI)**
α	G/G	269	363	1.00
	G/T	225	79	0.76 (0.68–1.62)
	T/T	49	37	1.18 (0.98–10.86)
Delta	G/G	68	465	1.00
	G/T	245	196	0.12 (0.05–1.01)
	T/T	39	13	0.65 (0.48–1.22)
Omicron BA.5	G/G	669	100	1.00
	G/T	94	124	8.83 (6.28–12.41)
	T/T	76	73	6.43 (4.38–9.43)

SARS-CoV-2, severe acute respiratory syndrome coronavirus 2; *VDR*, vitamin D receptor; OR, odds ratios; CI, confidence intervals.

**Table 4:** SARS-CoV-2 variants and *VDR* SNPs haplotype.

		α	Delta	Omicron
Haplotypes	Frequency	OR (95% CI)	OR (95% CI)	OR (95% CI)
TG	0.6319	1.00	9.01 (6.58–12.34)	0.45 (0.33–0.60)
CT	0.1677	0.93 (0.73–1.19)	2.29 (1.66–3.17)	0.50 (0.36–0.69)
CG	0.1267	0.81 (0.64–1.03)	0.81 (0.81–1.06)	1.17 (0.85–1.63)
TT	0.0737	1.27 (0.75–2.17)	1.27 (0.95–1.70)	1.20 (0.94–1.54)

SARS-CoV-2, severe acute respiratory syndrome coronavirus 2; *VDR*, vitamin D receptor; SNPs, single nucleotide polymorphisms; OR, odds ratios; CI, confidence intervals.

**Table 5:** Factors associated with deceased patients infected with COVID-19.

Factors		
Baseline Predictors	OR (95 % CI)	*p*-Value
Mean age ± SD	0.930 (0.912–0.948)	<0.001 *
ALT, IU/L	0.978 (0.969–0.988)	<0.001 *
HDL, mg/dL	1.034 (1.015–1.054)	0.001 *
LDL, mg/dL	1.018 (1.020–1.044)	<0.001 *
FBS, mg/dL	0.990 (0.987–0.998)	0.004 *
Uric acid, mg/dL	2.124 (1.815–2.486)	<0.001 *
Creatinine, mg/dL	0.064 (0.032–0.192)	<0.001 *
ESR, (mm/1st h)	0.971 (0.957–0.985)	0.001 *
25-hydroxyvitamin D, (ng/mL)	1.027 (1.007–1.046)	0.007 *
Real-time PCR Ct values	2.152 (1.967–2.354)	<0.001 *
SARS-CoV-2 variants	2.410 (1.847–3.145)	<0.001 *
*TaqI* rs731236 (CC)	0.318 (0.210–0.482)	<0.001 *
*BglI* rs739837 (TT)	4.870 (3.168–7.486)	<0.001 *

ALT, alanine aminotransferase; HDL, high-density lipoprotein; LDL, low-density lipoprotein; FBS, fasting blood glucose; ESR, erythrocyte sedimentation rate; Ct, cycle threshold; SARS-CoV-2, severe acute respiratory syndrome coronavirus 2; SD, standard deviation; OR, odds ratios; CI, confidence intervals. * Statistically significant (<0.05).

## Data Availability

All data generated or analyzed during this study are included in this article.
